# The Integration Preference of Sleeping Beauty at Non-TA Site Is Related to the Transposon End Sequences

**DOI:** 10.3389/fgene.2021.639125

**Published:** 2021-03-10

**Authors:** Yiting Zhou, Guangwei Ma, Jiawen Yang, Zenghong Gao, Yabin Guo

**Affiliations:** Guangdong Provincial Key Laboratory of Malignant Tumor Epigenetics and Gene Regulation, Medical Research Center, Sun Yat-sen Memorial Hospital, Sun Yat-sen University, Guangzhou, China

**Keywords:** transposon, non-TA sites, consensus sequence, sleeping beauty, integration

## Abstract

Recently, we proved that Sleeping Beauty (SB) transposon integrates into non-TA sites at a lower frequency. Here, we performed a further study on the non-TA integration of SB and showed that (1) SB can integrate into non-TA sites in HEK293T cells as well as in mouse cell lines; (2) Both the hyperactive transposase SB100X and the traditional SB11 catalyze integrations at non-TA sites; (3) The consensus sequence of the non-TA target sites only occurs at the opposite side of the sequenced junction between the transposon end and the genomic sequences, indicating that the integrations at non-TA sites are mainly aberrant integrations; and (4) The consensus sequence of the non-TA target sites is corresponding to the transposon end sequence. The consensus sequences changed following the changes of the transposon ends. This result indicated that the interaction between the SB transposon end and genomic DNA (gDNA) may be involved in the target site selection of the SB integrations at non-TA sites.

## Introduction

Sleeping Beauty (SB) transposon, a member of the Tc1/mariner family (Ivics et al., 1997), is the most widely used transposon genetic tool for gene therapy and the generation of genome-wide mutations (Dupuy et al., 2005; Starr et al., 2009; O’Donnell et al., 2012; Guo et al., 2016). Typically, DNA transposons have strong bias for their integration sites (Cary et al., 1989; Gangadharan et al., 2010; Guo et al., 2013). It was thought that SB, as well as other Tc1/mariner transposons, strictly integrates into TA dinucleotides (Ivics et al., 1997; Plasterk et al., 1999; Yant et al., 2005). However, this conclusion was based on the limited integration data before next generation sequencing (NGS) was widely used. Recently, we analyzed more than 2 million SB integration sites in mouse BaF3 cells and proved that SB could also integrate into non-TA sites at a frequency of ~1.4% (Guo et al., 2018). And further analysis suggested that SB might integrate into non-TA integration through an aberrant pathway (Guo et al., 2018). While reporting the non-canonical integration of SB for the first time, our study also raised several new questions: (1) given the integrations at non-TA sites were found in mouse cell lines, are there integration at non-TA sites in human cell lines? (2) The non-TA integrations we found were mediated by the hyperactive transposase version, SB100X (Mátés et al., 2009). Does the traditional SB11 transposase catalyze non-TA integration too? (3) Why does this consensus sequence only occur at one side of the integration site? and (4) We found that the consensus sequence flanking the integration site is the same as the sequence of the transposon ends, which was speculated the result of the interaction between the transposase and the target site, but is it possible that this phenomenon is the result of the interaction between the transposon end and the target site sequence?

To answer these questions, we performed integration assays in a human cell line, HEK293T, with both SB100X and SB11. We also constructed a series of plasmids with various combinations of mutated SB inverted repeat sequences (IR/DR) and found the preference of SB at non-TA sites is associated with the transposon end sequences.

## Materials and Methods

### Data Source

The raw sequencing data of the study of Chen et al. (2016) were obtained from the NCBI Short Read Archive.^1^ The accession number is SRX746204.

### Plasmid Construction

**Figure 1:**
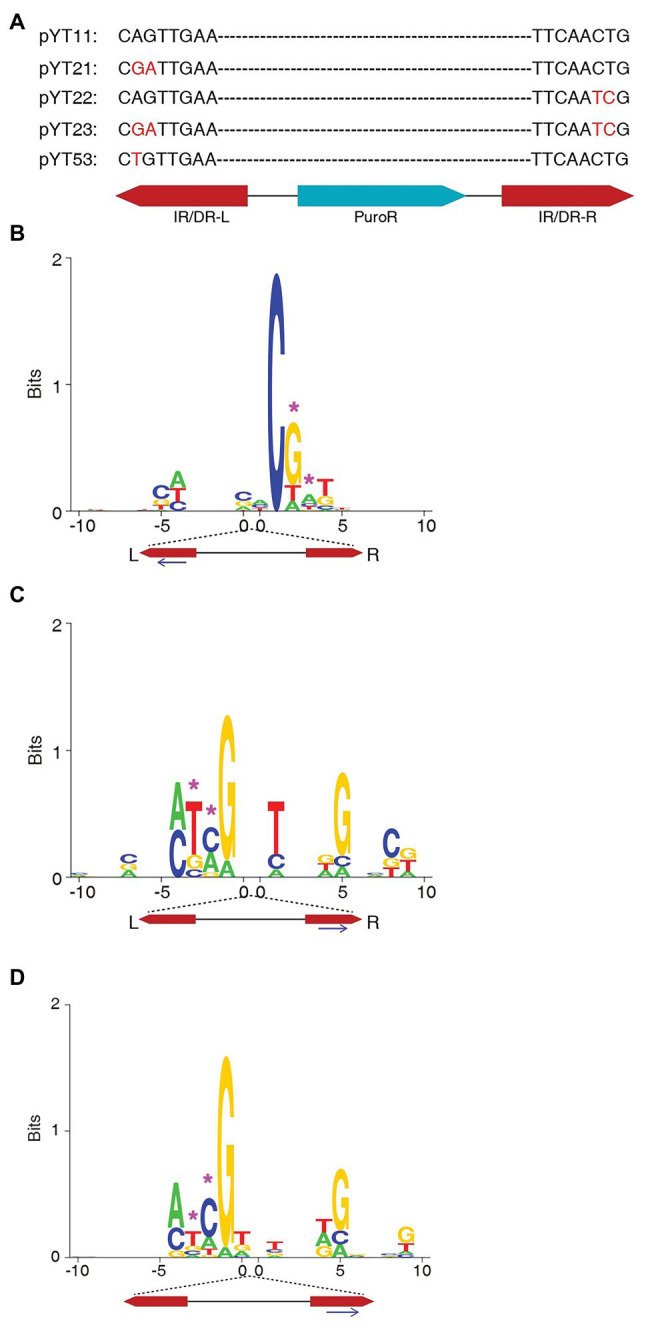
The target site consensus sequences of the non-TA integrations of the transposons with mutated IR/DR ends. **(A)** The design of mutations at the IR/DR ends. **(B)** The consensus sequences of non-TA target sites of pYT23 sequence from the left end. **(C)** The consensus sequences of non-TA target sites of pYT23 sequenced from the right side. **(D)** The consensus sequences of non-TA target sites of pYT23. The sequences from both left and right were combined. The position corresponding to the mutations was labeled with pink asterisks.

A puromycin resistance gene with promoter and polyA site was inserted between the IR/DRs of SB transposon, and this cassette was cloned into pUC19 backbone between HindIII and EcoRI restriction sites. pYT11 is the plasmid with classical SB ends. pYT21-23 and pYT53 have mutations at the IR/DR ends as described in the main text and Figure 1. The plasmids (1.25 μg) were transfected into HEK293T cells together with the transposase expression plasmids, SB100X or SB11 (1.25 μg), using Lipofectamine 2000/3000 (Thermo-Fisher) under the manufacturer’s protocol. After puromycin selection, cells were collected and genomic DNA (gDNA) samples were isolated. Then, ligation-mediated PCR (LM-PCR) assays were performed (Guo et al., 2016), and the amplicons were submitted for Illumina sequencing.

### Ligation-Mediated PCR

The gDNA samples were isolated using TIANamp gDNA Kit (TIANGEN). The LM-PCR assays were performed as described previously (Guo and Levin, 2010; Guo et al., 2016).

### Data Analysis

The sequencing data, including the data of this study and the data from SRA, were analyzed as previously described (Guo et al., 2018). Briefly, the NGS raw sequences were screened for the sequences containing the SB left or right end; the transposon end sequences were then trimmed and the sequences were aligned to the human genome (hg38) using Bowtie2 (Langmead and Salzberg, 2012). The output of Bowtie alignments were filtered using Perl scripts. The sequence logos were generated using an application, DNAlogo developed by our team (Guo et al., 2013, 2018; Chatterjee et al., 2014; https://www.biorxiv.org/content/10.1101/096933v2). The output PostScript (.ps) vector maps were converted to .pdf format in Adobe Illustrator.

## Results

### Non-TA Integration Sites Were Identified in Human Cells Using Both SB100X and SB11 Transposase

**Figure 2:**
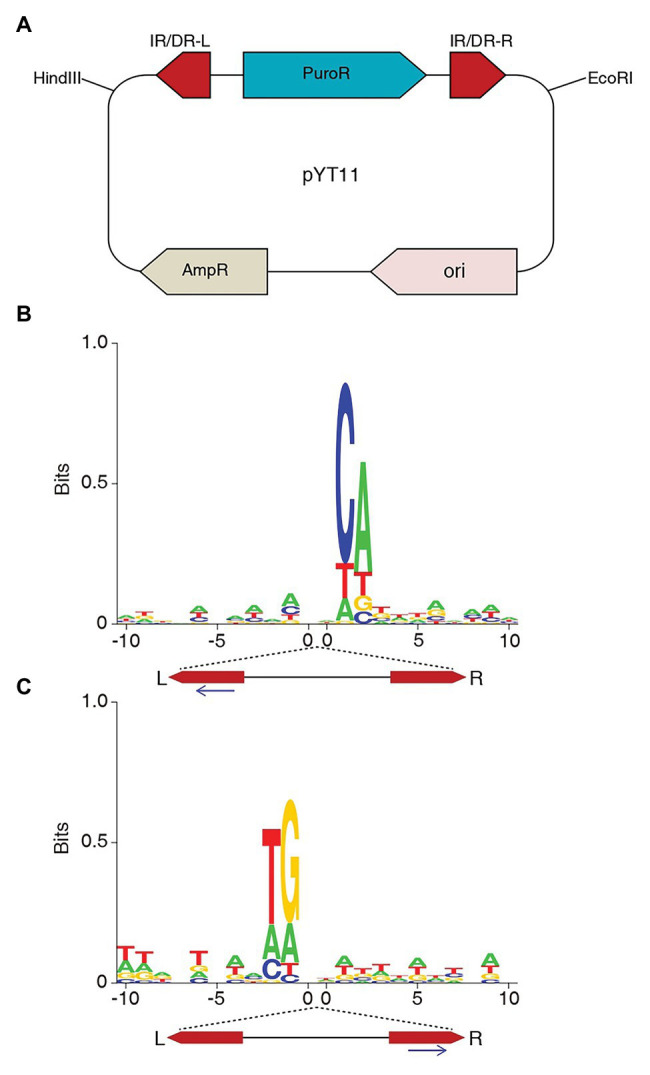
SB transposon integrates at non-TA sites in HEK293T cells. **(A)** The structure of plasmid pYT11, puromycin resistance gene flanked by the IR/DRs of SB. **(B)** The consensus sequences of the SB non-TA integrations sequenced from the left end. **(C)** The consensus sequences of the SB non-TA integrations sequenced from the right end.

We constructed a series of plasmids containing puromycin resistance gene flanked by the inverted repeat sequences of SB (IR/DR; Figure 2A). The plasmids were transfected into HEK293T cells with plasmids expressing SB100X or SB11. After puromycin screen, the cells were collected and gDNA samples were isolated. Then, LM-PCR and Illumina sequencing were performed to detect the integration sites.

After the sequences were aligned to the human genome, non-TA sites were identified (Table 1), which is similar to the observation in mouse BaF3 cells (Guo et al., 2018). We found non-TA integrations in the co-transfection of both SB100X and SB11 plasmids, indicating that SB11 can mediate integrations at non-TA sites as well as SB100X.

**Table 1:** Number of integrations at different dinucleotides.

	SB100X		SB11	
Dinucleotide	Left	Right	Left	Right
TA	29,748	27,740	3,731	460
CA	10	3	8	0
TG	7	5	5	2
TT	4	8	1	2
AA	5	9	0	0
GA	4	9	0	0
TC	8	1	3	0
AG	2	2	1	0
CT	7	3	3	0
GG	4	4	0	1
CC	4	5	3	2
AT	5	6	0	0
GT	2	2	1	1
AC	1	0	0	0
GC	7	1	3	1
CG	0	0	0	0
Total proportion of non-TA	29,818	27,798	3,759	469
	0.235%	0.209%	0.745%	1.919%

Usually, only the junctions between the SB left end and the genomic sequences were sequenced in the SB screening assays, because the left side gives better results in LM-PCR. Here, we sequenced both left and right junctions of SB integrations. Non-TA integrations were detected from both sides with similar proportions (Table 1). Notably, this does not mean that the non-TA junctions of left and right sides were from the same integrations, which was discussed in the next section.

### The Integrations at Non-TA Sites Are Mainly Aberrant

In our last study, we found a consensus sequence at the non-TA target sites, which is identical to the SB IR/DR end sequences. Here, we performed the same analysis with the integration data of this study. Figure 2B showed the similar pattern to what was found in our last study. The strong CA is corresponding to the CA/TG of SB ends. However, when we looked at the consensus sequence at the non-TA sites identified by sequencing the right end of SB, the consensus sequence occurred at the left side of the logo (Figure 2C). Interestingly, the consensus sequence is not fixed to the left or right side, but always occurs at the opposite side of the sequencing primers, which indicates that integrations at non-TA sites are mainly aberrant ones. The non-TA dinucleotides only occur at one side, whereas, those at the other side are still TA dinucleotides, thus were treated as canonical integrations when sequenced from the sides with TA dinucleotides. Although most of the integrations mediated by SB transposase have TA dinucleotides at both ends (Turchiano et al., 2014), there are still exceptions to notice in the studies of SB integration.

### The Consensus Sequence at the Non-TA Sites Is Corresponding to the Transposon End Sequences

To test whether the consensus sequence flanking the non-TA integration sites is related to the IR/DR sequences, we constructed plasmids with mutated IR/DR ends (Figure 1A). It is previously reported that the two nucleotides at the very end of the IR/DR are critical for SB transposition; mutation at the IR/DR ends almost abolish the transposition (Zayed et al., 2004). Therefore, we kept the first nucleotide unchanged and mutated the second and the third nucleotides from AG/CT to GA/TC (Figure 1A). The transposition efficiencies of SB with these mutated ends are similar to that of WT transposon in HEK293T cells (Supplementary Figure S1). Non-TA integrations were identified as well as in the integrations with native transposon end (Table 2) and it seems that the proportions of non-TA integrations of the transposons with mutated ends are higher than those with native ends.

**Table 2:** Number of integrations at different dinucleotides.

Dinucleotide	Left-mut	Right-mut
TA	39	73
CA	0	0
TG	3	1
TT	0	2
AA	0	1
GA	1	0
TC	2	2
AG	1	0
CT	0	1
GG	1	1
CC	0	0
AT	1	1
GT	0	0
AC	0	0
GC	1	1
CG	0	0
Total proportion of non-TA (%)	49	83
	1.77	1.68

The donor plasmid pYT23 has mutations at both inverted repeat sequences (IR/DR) ends.

The genomic sequences flanking integration sites were extracted and aligned. Surprisingly, the consensus sequences were all changed according to the changes of the transposon end sequences (Figures 1B,C). Since the number of total sites identified in this assay is small, to get a better view for the consensus sequence, the target sequences from both left and right sides were aligned together by the mutated ends (Figure 1D). Obviously, the consensus sequence (5' – ATCG3') perfectly reproduced the mutated transposon end.

**Figure 3:**
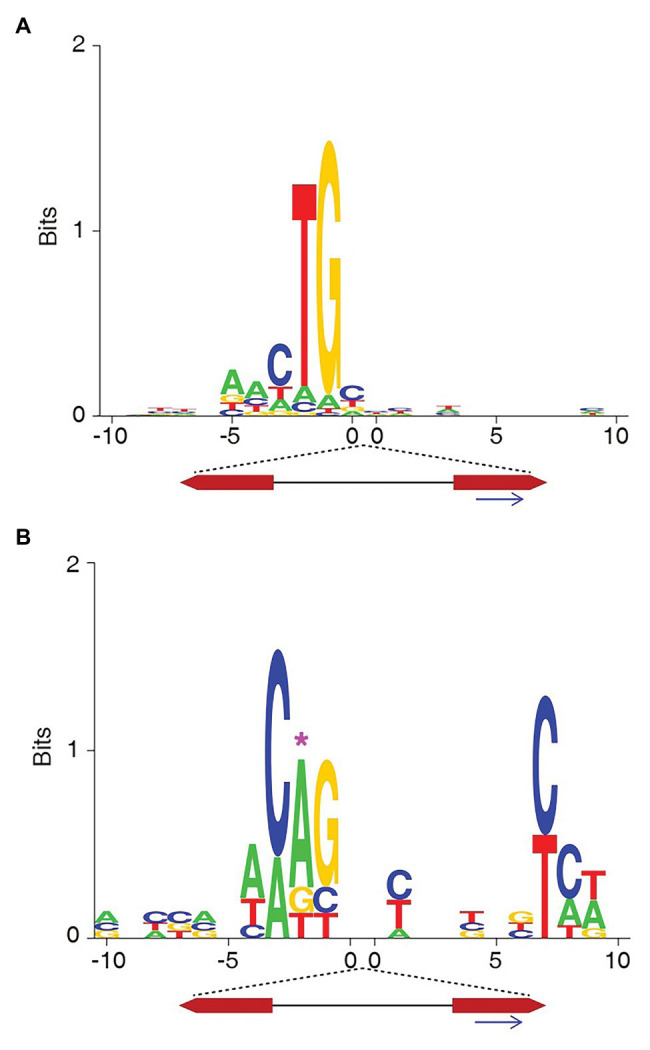
The target site consensus sequences of the non-TA integrations of the transposons with native or mutated IR/DR ends. **(A)** The consensus sequences of non-TA target sites of pYT22 sequenced from the left side (native end). **(B)** The consensus sequences of non-TA target sites of pYT53 sequenced from the left side. The position corresponding to the mutations was labeled with pink asterisk.

We also sequenced the left junction of the integrations of pYT22, which only has mutation at the right end. Figure 3A showed that the consensus sequence still reproduced the canonical transposon end (5' – ACTG3') as the previous observations.

The mutations in pYT21-23 are transitions. We also tried making transversion to the transposon end. pYT53 contains an A > T transversion at the second nucleotide of the SB left end (Figure 1A). Similarly, the consensus sequence at the target sites mimicked the transposon end (Figure 3B). These results indicate that the target site preference of SB at non-TA sites might be influenced by the transposon end sequences.

### The Non-TA Integration of SB Were Also Identified in Studies From Other Groups

**Figure 4:**
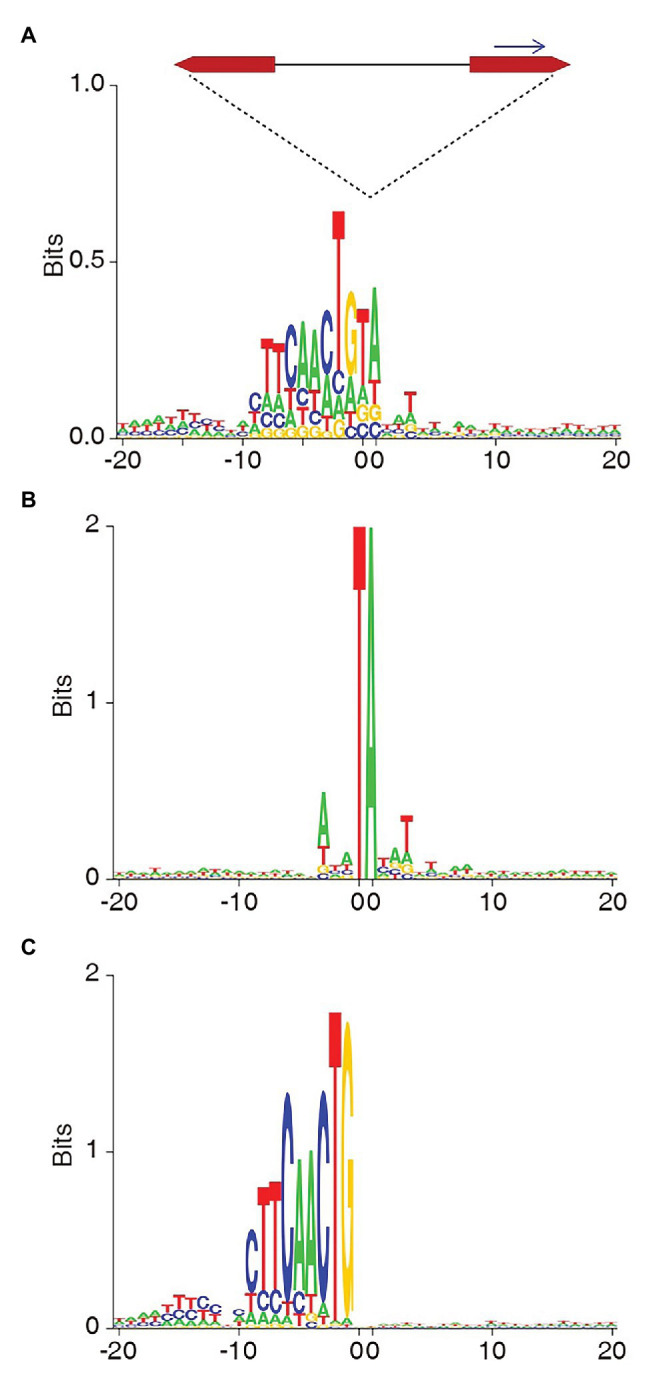
The target site consensus sequences of the total **(A)**, TA **(B)**, and non-TA **(C)** integrations identified from the raw next generation sequencing (NGS) data of the SB mutagenesis study by Chen et al. (2016).

Besides the studies of our team, Li et al. (2013) reported SB integrations in non-TA sites in 2013, and de Jong et al. (2014) reported the similar observation in 2014. In this study, we also searched several raw datasets from other SB mutagenesis studies. To our great surprise, we identified a large fraction of non-TA integrations from the raw data of a study on one of the study on recellularized human colon model by Chen et al. (2016). We identified 22,345 SB target positions from one of the raw dataset, SRR1634458, of which, more than half (54%) of the sites were not at TA dinucleotides (Table 3). The consensus sequence (Figure 4A) shows a moderate preference of TA at the TSD position and a strong pattern opposite to the sequenced side, which is distinct from the typical consensus sequence of SB target sites (Figure 4B). The consensus sequence of non-TA sites reproduced the transposon end perfectly as observed in our study, and its pattern is far stronger than those in our study, which could be due to the many more non-TA sites (Figure 4C). Of course, the authors of this article ignored these non-TA integrations following the canonical pipeline of data analysis. If the other half integrations at non-TA sites were considered, they might have got a more significant conclusion.

**Table 3:** The non-TA integrations from one of the previous Sleeping Beauty (SB) mutagenesis study (Chen et al., 2016).

Target	Count	Proportion (%)
TA	19,084	46.06
AT	2,139	5.16
TG	2038	4.91
TC	1913	4.61
CA	1903	4.59
AG	1798	4.33
TT	1778	4.29
CC	1,659	4.00
AA	1,526	3.68
AC	1,395	3.36
GG	1,374	3.31
CT	1,281	3.09
GA	1,271	3.06
GC	1,087	2.62
GT	1,056	2.54
CG	127	0.30
total non-TA	41,429	100
	22,345	53.93

## Discussion

In our last study, we reported the SB integrations at non-TA dinucleotides catalyzed by SB100X in mouse cells (Guo et al., 2018). Here, we performed integration assays in human HEK293T cells with both SB100X and the traditional SB11 transposase. Our results showed that both SB100X and SB11 can mediate non-TA integration in mouse cells and human cells, indicating that non-TA integrations keep happening in typical SB integrations assays and attentions might need to be paid by researchers.

It is shocking that there were so many (54%) non-TA integrations in the study of Chen et al. (2016). Although we cannot speculate the reason for such a high proportion of non-TA integrations in their experiments, these findings may suggest that non-TA integration is far more common than people have thought and its proportion can be fairly high under certain circumstances.

Geurts reported that the TA sites in the mouse genome are not equally favored by SB targets and more than half of the insertions were clustered in the ~10% hot TA sites (Geurts et al., 2006). The consensus sequence of the non-TA sites found in our studies is not similar to the sequences at those hot spots and may be hard for the pre-integration complex (PIC) to access, which could be partially account for the low frequency of the non-TA integrations.

**Figure 5:**
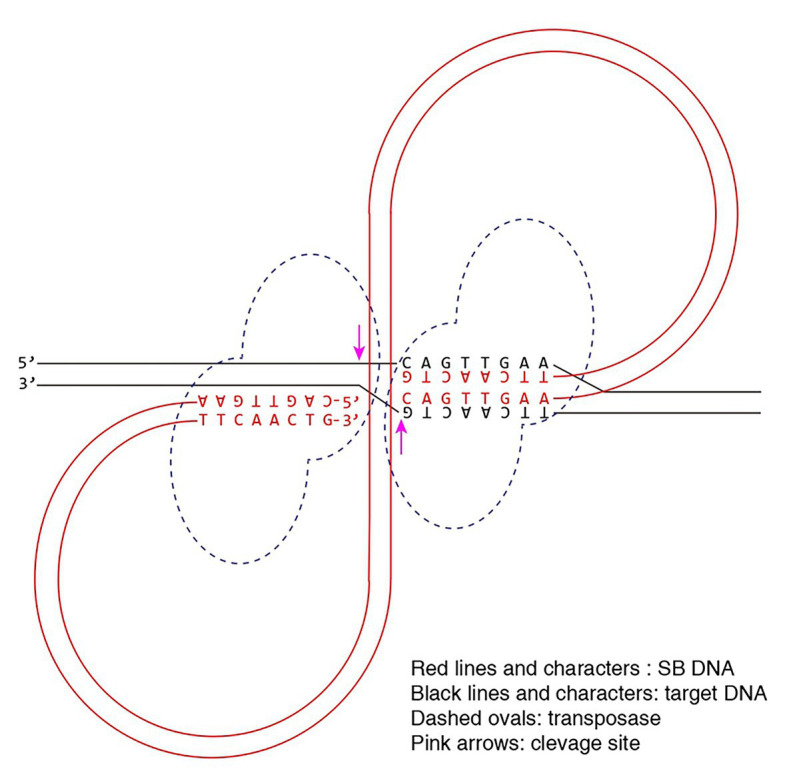
Model for the alternative integration mechanism of Sleeping Beauty transposon. The pre-integration complex (PIC) recognizes the target site *via* the interaction between the transposon DNA and the target DNA.

The consistency of the consensus sequence at the non-TA sites and the transposon end sequence is fascinating. In our last study, following the suggestion of the reviewers’, we hypothesized that the consensus sequence is the result of the interaction between the transposase and the target DNA (Guo et al., 2018). However, the current study seems indicate that the consensus sequence is due to the interaction between the transposon end DNA and the target DNA. Therefore, we hypothesize that besides the canonical integration mechanism that relies on the interaction between transposase dimer/tetramer and target DNA, including TA dinucleotide, there might be an *alternative integration mechanism* for SB transposon that relies on the interaction between one of the transposon ends and the target DNA, resulting in asymmetric and aberrant integrations (Figure 5). Notably, the sequences at the target site are not exactly the consensus sequence (Guo et al., 2018), and the more they are similar to the consensus sequence, the stronger the interactions would be. Although the similarity between the consensus sequence and the SB ends leads people to imagine the possibility of homologous recombination, it actually is unlikely, which has been discussed previously (Guo et al., 2018).

Previous study showed that the excisions of SB are influenced by the borders of the transposon and the flanking sequences (Liu et al., 2004). It is possible that the different pre-integration SB transposon ends are different between the non-TA integrations and the canonical integrations, so that the non-canonical integrations are a result of non-canonical excision, which is to be answered by the future studies. One limitation of this study is that we only tested the SB integrations in one cell line, the HEK293T, and the cases in more other cell lines are still to be tested.

To our knowledge, we are the first to report that the transposon integration preference is not only determined by the transposase, but also can be influenced by the transposon end sequences. Now, deep sequencing provides good opportunity for studying the asymmetric pattern of SB integration. We believe that our results can bring new ideas to the mechanism study on the target site determination of transposons. Finally, we again suggest that researchers should not ignore the non-TA integrations in the data analyses of SB mutagenesis, and more importantly they should consider the possibility of non-TA insertions in gene therapies for the safety purpose.

## Conclusion

The integrations of SB transposon at non-TA sites can be catalyzed by either SB11 or SB100X in either human or mouse cells. The interaction between the SB transposon end and gDNA may be involved in the target site selection of the SB integrations at non-TA sites.

## Data Availability Statement

The raw sequencing data of the study of Chen et al. (2016) were obtained from the NCBI Short Read Archive (http://www.ncbi.nlm.nih.gov/sra). The accession number is SRX746204.

## Author Contributions

YG conceived the idea for the project. YZ and YG designed the experiments and wrote the manuscript. YZ and GM performed the experiments. YZ, JY, ZG, and YG analyzed the data. All authors contributed to the article and approved the submitted version.

### Conflict of Interest

The authors declare that the research was conducted in the absence of any commercial or financial relationships that could be construed as a potential conflict of interest.

## Footnotes

^1^http://www.ncbi.nlm.nih.gov/sra

## Abbreviations

SB: Sleeping beauty
IRDR: Inverted repeat direct repeat
NGS: Next generation sequencing
LM-PCR: Ligation-mediated PCR
TSD: Target site duplication
