# Supplementary figures and images for: The Integration Preference of Sleeping Beauty at Non-TA Site Is Related to the Transposon End Sequences
Source: Front Genet. 2021 Mar 10;12:639125. doi: 10.3389/fgene.2021.639125 (PMC7987939; doi:10.3389/fgene.2021.639125)

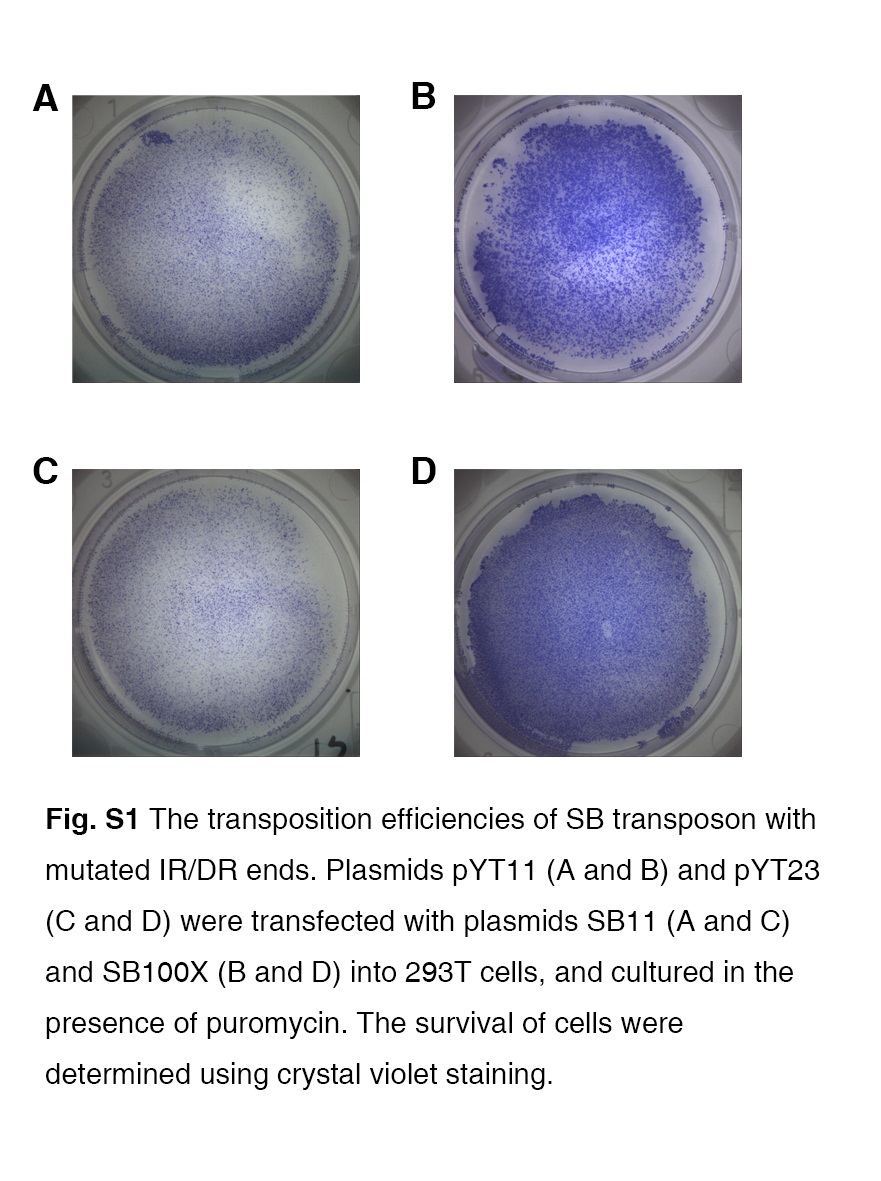

Supplement: Supplementary file 1 [file Image_1.jpeg]
